# Variation under domestication in animal models: the case of the Mexican axolotl

**DOI:** 10.1186/s12864-020-07248-9

**Published:** 2020-11-23

**Authors:** María Torres-Sánchez

**Affiliations:** 1 Department of Neuroscience, Spinal Cord and Brain Injury Research Center & Ambystoma Genetic Stock Center, University of Kentucky, Lexington, KY 40536 USA; 2 Present address: Department of Biology, University of Florida, Gainesville, FL 32611-8525 USA

**Keywords:** Artificial selection, Protein-coding genetic variation, Leucism, Reproducibility, Single nucleotide variants, Transcriptomics

## Abstract

**Background:**

Species adaptation to laboratory conditions is a special case of domestication that has modified model organisms phenotypically and genetically. The characterisation of these changes is crucial to understand how this variation can affect the outcome of biological experiments. Yet despite the wide use of laboratory animals in biological research, knowledge of the genetic diversity within and between different strains and populations of some animal models is still scarce. This is particularly the case of the Mexican axolotl, which has been bred in captivity since 1864.

**Results:**

Using gene expression data from nine different projects, nucleotide sequence variants were characterised, and distinctive genetic background of the experimental specimens was uncovered. This study provides a catalogue of thousands of nucleotide variants along predicted protein-coding genes, while identifying genome-wide differences between pigment phenotypes in laboratory populations.

**Conclusions:**

Awareness of the genetic variation could guide a better experimental design while helping to develop molecular tools for monitoring genetic diversity and studying gene functions in laboratory axolotls. Overall, this study highlights the cross-taxa utility that transcriptomic data might have to assess the genetic variation of the experimental specimens, which might help to shorten the journey towards reproducible research.

## Background

Biological variation is inherent to all living organisms. Animals that are used in research experiments present different levels of phenotypic and genetic variation not only as a result of their natural evolutionary history but, in some cases, also as a consequence of domestication processes. Species adaptation for human use, namely domestication, has modified captive populations of many species when compared to their wild counterparts. Being analogous to natural selection, domestication has greatly expanded our understanding of biological variation and evolution since Darwin [1, 2] and Wallace [3], offering a framework to study different biological traits [4, 5]. By means of artificial selection, unusual phenotypes and mutants can be propagated in captivity expanding the phenotypic variation of the domesticated populations in relation to their free-living conspecifics [6]. In contrast, genetic variation is typically skewed and reduced in domesticated populations [7], since they are generally established from relatively few individuals of the natural source populations leading to an underrepresentation of the total genetic variation. Through the years of captive breeding, animal import, genetic drift, and non-random mating, the genetic structure of captive populations can be altered increasing the differences both between wild and domesticated animals, and among different captive populations [4, 7, 8].

Laboratory animals constitute a special case of domestication being suited for research purposes. This special domestication process has also changed laboratory animals phenotypically and genetically. Despite the major impact of domestication, this variation is not well documented in some cases. The characterisation of the genetic variation of laboratory animals is crucial to understand the genomic background of the specimens used in biological experiments, which can influence greatly the experimental outcome of the studies [8–14]. Accordingly, knowledge about the genetic variation of laboratory specimens is required to characterise differences among populations, to define strains, and, overall, to better ensure the reproducibility of any experiment.

The Mexican axolotl, *Ambystoma mexicanum* (Shaw and Nodder, 1798) is the animal model with the deepest laboratory pedigree. Axolotls, native to the Xochimilco lake system, México, are paedomorphic salamanders that have been bred in captivity since 1864 [15]. Paradoxically, while laboratory populations are flourishing around the globe, axolotls are critically endangered in the wild and, despite conservation efforts, the source populations might sadly get extinct [16, 17]. Most of the current laboratory populations trace their ancestry to the 34 animals that once comprised the first domestic population in Paris, France [15, 17]. Those animals presented typical dark green-to-grey axolotl coloration (commonly referred to as wild-type) with the lone exception of a white-coloured male axolotl [18]. This white pigment or leucistic phenotype emerges from the absence of melanocytes and is determined in axolotls by a variant of the gene endothelin 3, *edn3* [19]. From the progeny of that white axolotl a historic captive population was created in 1935 and its descendants are nowadays housed by the Ambystoma Genetic Stock Center (AGSC, [17]). White axolotls that are extremely rare in the wild are thriving in laboratory populations being propagated by artificial selection, which potentially has modified their genetic background beyond the causal phenotypic mutation.

Despite the extensive use of axolotls in biological research, the genetic diversity of laboratory populations, including the pigment phenotypes, has not been previously characterised. Given the complex domestication history of axolotls, great genetic variation and structure among captive populations could be expected. Here, I tested whether axolotls used in foregoing gene expression experiments are genetically distinct with four main objectives: i) discover trends of selection along predicted protein-coding genes potentially related to artificial selection, ii) uncover genome-wide differences between pigment phenotypes from the same laboratory population, iii) identify private variants of the only wild-caught axolotl from Xochimilco with transcriptomic data, and iv) discover bias in the genetic background of the specimens used in each project. More generally, the research approach followed here aims to highlight that transcriptomic data can be used not only to describe gene expression patterns but also to disentangle the genomic background of the study specimens, and, thereby, to account for that vital source of variation.

## Results

### General patterns of protein-coding variation

DNA sequence variants were identified for 146 samples that were sequenced by RNA-Seq (see Supplementary Table S1 for further information about the samples). A total of 585,766 variants were found along 22,022 protein-coding sequences, representing 94.72% of the predicted protein-coding sequences of the *A. mexicanum* genome assembly. A total of 1229 protein-coding sequences did not contain any variants in this study. The variants were categorized into two groups: i) insertions and deletions of bases (INDELs, 128,504, 21.94%), and ii) single nucleotide variants (3289 multiallelic and 453,973 bi-allelic variants, 0.56 and 77.5%, respectively). Bi-allelic, single nucleotide variants (SNVs) consisted of 282,603 transitions (62.25%) and 171,370 transversions (37.75%; see Supplementary Table S3). SNVs were also classified using their minor allele frequency (MAF), where 251,934 variants presented a MAF > 0.01 (55.5%, for further information about number of SNVs at different MAF values, see Supplementary Table S4).

**Fig. 1:**
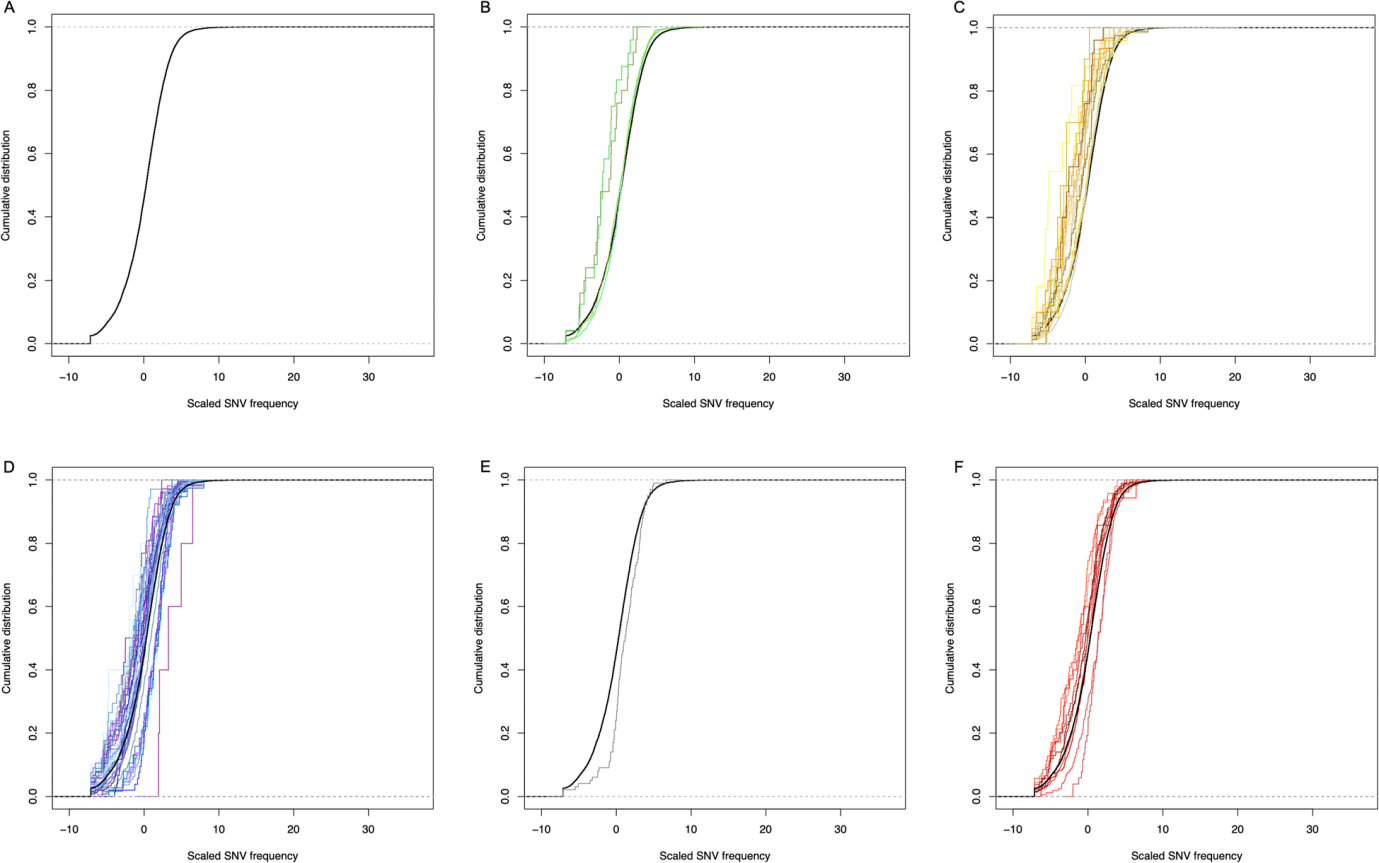
Representation of SNV frequency distribution and deviated distribution of sequence sets classified by PANTHER annotations. **a** Empirical cumulative distribution of SNV frequency scaled by the overall SNV frequency. The value 0 indicates the mean SNV frequency (7.13 SNVs per kilobase) and was used to rank the sequences before the graphical representation. **b–f** The graphs show the results of the PANTHER statistical enrichment test representing the SNV frequency distribution of sequence sets annotated with a annotation term that deviated significantly from the overall distribution. Distribution curves of each graph are color-coded by the annotation set: (**b**) GO-Slim Biological Process, **c** GO-Slim Cellular Component, **d** GO-Slim Molecular Function, **e** Pathways, and **f** Protein Class. Distribution curves to the left of the overall SNV frequency distribution (in black color) represent a set of sequences annotated under an annotation term that have fewer SNVs than expected while distribution curves to the right of the overall SNV frequency distribution symbolise a set of sequences with more SNVs than expected

The mean frequency of protein-coding sequence variation from the identified SNVs was 7.13 SNVs per kilobase, Kb (see the sequences cumulative distribution based on their scaled SNVs frequency in the Fig. 1a). A total of 17 predicted genes had a SNV frequency > 20 per Kb (*agrin, mgc83164, sbno2, cbx2, ulk4, pr* [AMEXTC_0340000102199_PR]*, fanca, loc104496260, ube3b, loc104369368, loc108695830, ezh1*, mRNA00976, mRNA00719, AMEXTC_0340000073769_hypothetical, and AMEXTC_0340000214929_hypothetical). The number of SNVs may reflect the functional importance of a protein, and the strength and mode of selection that acts to maintain or diversify the underlying coding sequence. PANTHER enrichment tests identified 33 significantly enriched annotation terms, which were supported by at least a set of 100 sequences. Those 33 set of sequences classified under the same term yielded SNV frequency value distributions that deviated significantly from the overall distribution (see Fig. 1b–f and Supplementary Table S5). Three terms were enriched by genes with higher than expected SNV frequency values; these included the Protein Class term *G-protein modulator* (*N* = 172, false discovery rate [FDR] *p* value = 2.70E-04) and Pathways term *PDGF signalling pathway* (*N* = 100, FDR *p* value = 1.03E-02). In contrast, the other 30 terms were enriched by genes with fewer SNVs than expected. Many of these terms were significantly enriched by genes that encode proteins with binding functions, including DNA-binding functions, associated with transcription and gene expression (e.g. Molecular Function gene ontology, GO, terms: *nucleic acid binding*, *N* = 838, FDR *p* value = 5.88E-06; and *transcription regulatory activity, N* = 506, FDR *p* value = 2.68E-02; see Supplementary Table S5). These results suggest a similar type of selection on sequences that are annotated with the same term.

### Diversity and relationships among RNA-Seq samples

For each RNA-Seq sample, SNVs were counted and compared to the total number of SNVs identified across all samples. On average, 64.30% of the total number of SNVs was represented in any given RNA-Seq sample (see SNVs information for each sample in the Supplementary Table S1). SNVs with non-reference alleles were used to quantify genetic variability among samples by computing heterozygosity ratios. The mean heterozygosity ratio for all samples was 1.42, with values ranging from 0.51 to 5.3 across samples. Mean heterozygosity ratios were also calculated for each project. The project (PRJNA354434) that sequenced multiple tissue samples from a single wild-caught animal had the lowest ratio with a mean of 0.59, followed by the project PRJNA306100 with a mean ration of 0.64, while the project PRJNA378982 had the highest ratio with a mean of 2.69.

**Fig. 2:**
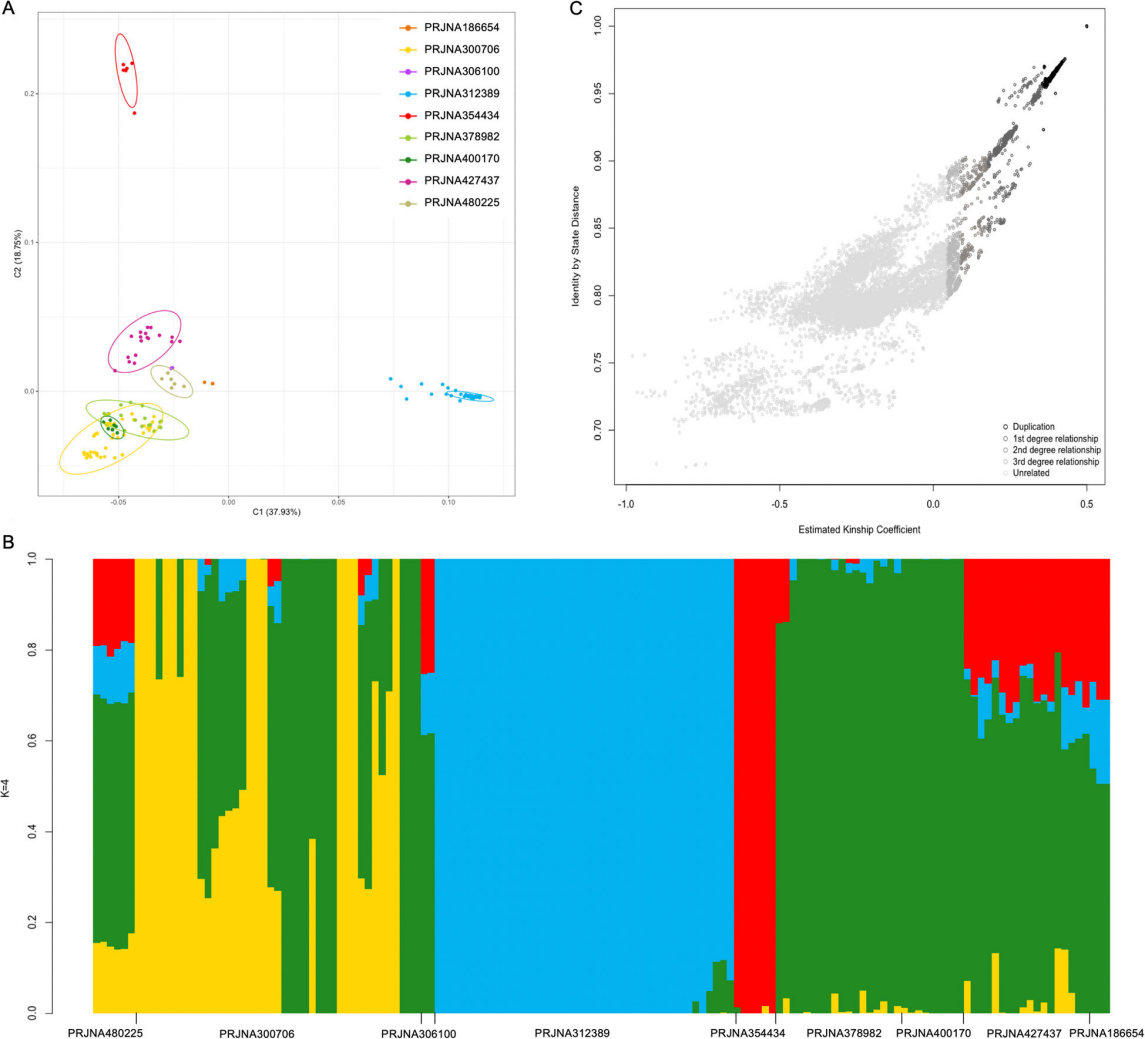
Sample variation. **a** Variance analysis of samples using multidimensional scaling. The plot shows dimensions one and two. Samples from each project are symbolized by different colors and plotted in reduced multidimensional space. Samples from projects represented primarily by one of the four Admixture clusters were represented with the same Admixture cluster color. **b** For each project, cluster proportions of Admixture analysis are displayed. **c** Correlation between Identity by State (IBS) and Identity by Descendant (IBD), as measured by the Kinship Coefficient. Sample relatedness levels are color coded from twins/duplications = black to pair of unrelated samples = light grey

Genetic differences among samples were further explored by measuring genetic distance and by using multidimensional scaling analysis, MDS (see Fig. 2a and Supplementary Fig. S1 for a graphical representation of the MDS coordinates, and Supplementary Table S6 for percentage of variance explained by each of the coordinate/dimension). The first three coordinates explained 65.24% of the genetic variance among samples and identified three different clusters that grouped their own samples (PRJNA312389, PRJNA354434 and PRJNA427437). Samples within other projects were clustered together by coordinate six (PRJNA186654 and PRJNA306100), coordinate seven (PRJNA480225), and coordinate nine (PRJNA400170), with each of these coordinates explaining 5.75, 4.97, and 3.7% of the genetic variance, respectively. To characterize the nature of the genetic differences among samples, Admixture and relatedness analyses were performed. The maximum likelihood number of ancestral clusters was four (see Supplementary Fig. S2). The proportional representation of these clusters varied among samples (Fig. 2b). Samples from three projects (the first two already grouped by the MDS analysis, PRJNA312389, PRJNA354434, and PRJNA400170) were represented primarily by one of the four clusters. In terms of relatedness, pairwise comparisons revealed high levels of kinship among samples. The six wild-caught samples (project PRJNA354434) were identified as duplicates (samples from the same organism, technical replicates, 0.354 inferred kinship coefficient or higher). Also, duplicates, first, second and third-degree relationships (kinship coefficients ranging from 0.354 to 0.177, from 0.177 to 0.0884, and from 0.0884 to 0.0442, respectively) were identified for same samples within projects PRJNA480225, PRJNA300706, PRJNA312389, PRJNA378982, PRJNA400170, PRJNA186654. Only all the samples within projects PRJNA306100 and PRJNA427437 showed kinship coefficients lower than 0.0442, which is typical of unrelated samples. Among projects, the majority of the pairwise relationships showed a negative estimated kinship coefficient, which is suggestive of heterogeneity and genetic structure (see Fig. 2c and Supplementary Fig. S3).

The genetic diversity and relationship analyses described above were performed in an unbiased way, without prior information about the sample project. Fixation indexes (F_ST_) as a measure of population differentiation were calculated for each pair of projects to further assess population differences among projects, and to potentially identify projects that used animals from the same population. The estimated F_ST_ values ranged from 0.087 (F_ST_ between PRJNA300706 and PRJNA378982) to 0.664 (F_ST_ between PRJNA306100 and PRJNA354434, see Supplementary Table S7). The highest F_ST_ values were estimated for all projects compared against PRJNA354434, which used wild-caught axolotl samples, followed by the values against the project PRJNA306100. This indicates that the wild-caught axolotl samples were the most genetically distinct, sharing less genetic variation with samples from all other projects. The projects PRJNA480225, PRNJA300706, PRJNA378982, PRJNA427437, and PRJNA400170 (with the exception of the pairwise value between PRJNA 400170 and PRJNA480225, F_ST_ = 0.262) shared values smaller than 0.2, which might indicate that animals used in those projects came from the same interbreeding laboratory population.

### Pigment phenotypes comparison

**Fig. 3:**
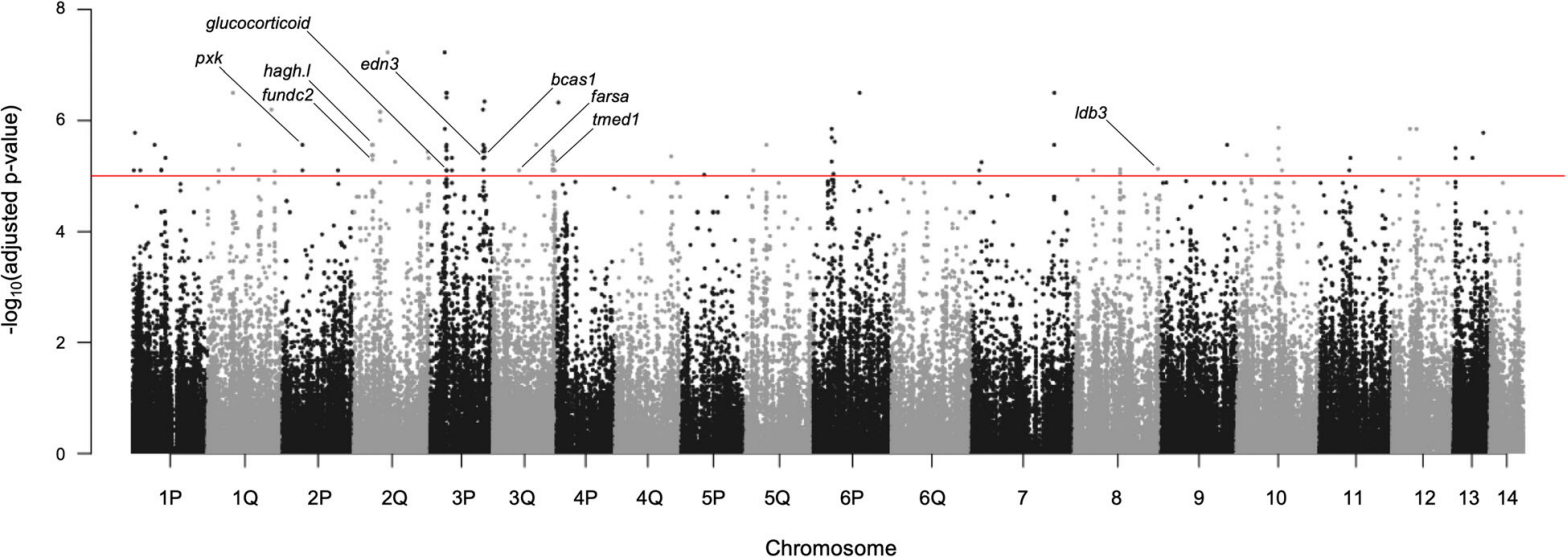
SNVs associated with the pigment phenotype. The Manhattan plot represents the results of the association analysis between white and wild/wild-type samples. Variant-phenotype associations were identified using an adjusted *p*-value <1e-5 threshold, which is represented by the red line. Sequences name tags highlight identified variants that caused unequivocally a change in the amino-acid sequence

The RNA-Seq projects isolated tissue mainly from either wild-type or white axolotls. Using a selection of samples from the same interbreeding laboratory population (see Supplementary Table S2 for detailed information of the samples and Supplementary Fig. S4 for a representation of the likelihood of the Admixture analysis for this sample subset), associations between variants of the gene predictions and the pigment phenotypes were identified by genome-wide association analysis using the threshold of adjusted *p*-value <1e-5 (see Fig. 3 and Supplementary Table S8). A total of 104 SNVs were identified in 63 gene predictions along with the gene *edn3.* Most of the genes (40%) with significantly-associated variants for pigment phenotype were located to chromosomes 3 and 1 (Chr3: *edn3, bcas1, gmeb2, amextc_0340000052082_hypothetical, nsfl1c, dnm2, kcnmb1.l, loc108702636, glucocorticoid, loc102357877, mapre1.l, rad50, tmed1,* and *farsa*; Chr1: *mydgf, fbn3, naa38, loc104535489, syt11, loc100619372, nuf2.l, enpp4, lipe, rnf11.l,* and *top1.2*). Variants in several genes across all chromosomes except for Chr14 were identified. Two variants were found in genes that have not been mapped to the chromosome assembly (*sfrp2* and *loc108717189*). The identification of SNVs that are associated with the white pigment phenotype but not physically associated with *edn3* reveals genomic differences between white and wild-type axolotls. Genes bearing SNVs associated to wild-type/white phenotypes were generally found more than 10 megabases (Mb) apart, however some genes in the short arm of Chr3 (Chr3P) and a pair of genes in Chr6 and Chr7 were found more closely. A total of 16 variants caused unequivocally a change in the amino-acid sequence of their gene predictions (*bcas1*, *edn3*, *farsa*, *fundc2*, *glucocorticoid*, *hagh.l*, *ldb3*, *loc103760181*, *loc106732794*, *loc108702636*, *pxk*, and *tmed1*; see Supplementary Table S8) resulting in a different phenotype, at least in the amino acid profile, between wild-type and white axolotls beyond their skin coloration.

### Private variation of the wild-caught axolotl

Using sequence data from project PRJNA354434, which sequenced RNA from a wild-caught axolotl, 25,825 SNVs (5.69% of the total SNVs) distributed across 10,639 protein-coding genes were identified as unique to this project. A total of five gene predictions showed more than 20 wild-caught private SNVs (*loc108701391*, *tropomyosin*, *ncaph2*, *fructose-bisphosphate*, and *cnp*). The frequency of private, wild-specific SNVs among chromosomes was uneven, with significantly more SNVs observed for genes on Chr2 and Chr3, and significantly fewer observed for chromosomes 4, 7, 9, 12, and 13 (see Supplementary Table S9). These variants have been plausibly lost in domesticated axolotls.

## Discussion

Species adaptation for human use has changed the genetics of the domesticated populations when compared to their wild counterparts. Animal models are domesticated species adapted to the laboratory life for research purposes. This special domestication process has also changed animal laboratory populations phenotypically and genetically. This variation is not well documented for example in the Mexican axolotl. Axolotls have been bred in captivity for the last 155 years [15]. While the complex history of contemporary laboratory populations has been previously described [17, 19], very little is known about existing levels of genetic variation. In this study, reads from 146 RNA-Seq samples, collected from different research projects, were aligned to the gene predictions of the axolotl genome, and DNA sequence variants were identified. This study unravelled a genetic diversity bias among specimens used in different experimental studies and, also, different genetic background between axolotl pigment phenotypes. The results presented here will aid researchers not only in monitoring axolotl genetic variation within laboratory populations, but also in refining future studies and experiments. Particular attention to the genetic variation described here should be given when performing gene editing experiments to distinguish the treatment effects and the specimen genetic backgrounds [20]. Additionally, this study yields a prioritized list of candidate genes for further studying protein-coding gene evolution under domestication processes. Ultimately, this research could guide other transcriptomic studies to not only describe gene expression patterns but also to uncover the genetic variation of the study specimens of any species. This should be particularly the case of model organisms, for which this information could help to disentangle the genetic imprints of domestication and to establish the foundations for more reproducible research.

Consistent with patterns of genetic variation described for other species [21–23], the most prevalent type of sequence variant identified among axolotl RNA-Seq datasets were biallelic SNVs with a transition/transversion ratio of 1.65 (around 60 and 40% of the SNVs, respectively). Common and rare variants (MAF > 0.01 or 0.05) were also in agreement with previous population genetics studies showing a high percentage of rare variants in coding regions [24]. Animal laboratory populations are usually small and under founder effects and genetic drift processes, which lead to inbred and/or considerable divergent laboratory populations [14]. Despite this, relatively high levels of heterozygosity were described in this study that could have reached by crosses between different captive populations and/or hybridization with closely related species. Introgressive hybridization from a tiger salamander (*Ambystoma tigrinum* Green, 1825) into axolotl laboratory populations was undertaken in 1962, and tiger salamander genetic segments persist today among AGSC axolotls [19]. Before further discussion of the results, it has to be mentioned two primary caveats noted in classifying SNVs mined from RNA-Seq datasets using allele frequencies. Some of the RNA-Seq samples (for example project PRJNA480225 samples) that were included in the analysis contained RNA from more than one individual, and thereby the estimates of heterozygosity may reflect interindividual genetic variation and not true heterozygosity. Second, even for samples that were created from single individuals, both alleles at a locus may not be simultaneously expressed [25]. Despite the plausible missing genotype of loci related to transcriptome data, on average, more than 60% of the SNVs were discovered within each RNA-Seq sample, allowing to use this reduce representation technique for characterizing genetic diversity [26].

SNVs identified in this study were not randomly distributed across gene predictions. Many studies have shown that SNVs can vary among genes because of differences in the ages of genes and evolutionary processes like selection [27, 28]. It is widely recognized that evolutionary processes can shape genetic diversity of a given gene and genes under the same function or structural constrain [29–31], and, here, annotation terms that were significantly enriched by genes that showed similar deviations in SNV frequency were identified. Axolotl genes with relatively fewer SNVs enriched annotation terms that were associated with highly conserved and essential functions mainly related to *nucleic acid binding*, including transcriptional regulation. Those genes may be similarly constrained plausibly by the same relative strength of purifying selection. Genes with relatively more SNVs were associated with *G-protein modulator* and *PDGF signalling pathway* and could be under more relaxed selection. The functional significance of SNVs discovered in this study await further study as both non-synonymous and synonymous SNVs can affect gene functions, as well as splicing, folding, mRNA stability, gene expression, and consequently, the associated biological processes [32–34].

Beyond functional constraints and natural selection, it is possible that genes with variable numbers of SNVs may present genomic signatures of artificial selection, drift, and introgressions related to election of specific phenotypes in the course of axolotl domestication. During early axolotl domestication, white axolotl lineages were propagated independently from two lineages and subsequently integrated into the AGSC population [17]. Giving that the white pigment phenotype is a recessive trait rarely seen in nature but extremely common in captive populations, the propagation of white individuals was undoubtedly accomplished by non-random mating to maintain white alleles. Because white axolotls are preferentially mated to each other and not to wild-type individuals, unique polymorphisms likely accrued between these strains, including SNVs that are not linked with *edn3,* the causative gene for the white phenotype [19]*.* The genetic association analysis supports this idea having identified SNVs throughout the axolotl genome associated to RNA-Seq samples from axolotls with different pigment phenotypes. Likewise, some of these variants changed the amino-acid sequences of the predicted proteins and, accordingly, the phenotype of the axolotls beyond pigmentation. A genome-wide effect of domestication could be also supported by the discovery of SNVs that were unique to the Xochimilco axolotl RNA samples. Presumably, these SNVs were lost during captive breeding. However, there is need to test additional axolotl samples collected from nature and additional laboratory populations to more extensively characterise the variation of the Xochimilco and captive populations. Likewise, historical samples stored in collections and museums would be required to thoroughly describe axolotl domestication process. Given the impoverished state of the Xochimilco axolotl population, critically endangered and having suffered dramatic reductions in population size for many years [16, 17], the wild populations could no longer reflect the variation of the wild source from which domestic populations arose. Breeding management strategies have shaped genetic variation of laboratory animal populations, causing in some cases large genetic differences plausibly not only between natural and laboratory animals, but also among laboratory strains, that can affect experimental reproducibility, including studies of gene expression [7, 8, 35]. The genetic differences found here need to be further investigated to address whether this variation is affecting biological processes.

Across projects, axolotls showed different levels of genetic variation. One component of this variation is likely explained by the experimental design strategy that these projects employed. Genetic differences among samples revealed two principal experimental design strategies: experiments with siblings from probably the same hatch (e.g. PRJNA312389 and PRJNA480225) and experiments that used non-siblings (e.g. PRJNA427437). RNA samples from projects that used first/second degree related axolotls, or as in the case of project PRJNA354434, where all the RNA samples were obtained from a single individual, were clustered together by Admixture analysis. Giving the nature of these Admixture clusters, sample ancestries were misled by sample relatedness. The Admixture analysis performed here provided a complementary insight of genetic variation, allowing to further describe variation among samples and projects rather than divergence and admixture stratification [36]. The RNA samples from the Xochimilco axolotl were the most different in this study and the second most dissimilar samples were from the project PRJNA306100, which used axolotls from a population that may have descended from a non-AGSC lineage of the original Parisian captive population [15]. Beyond the sibling vs. non-sibling experimental design strategy, I highlighted that projects that disclosed the sample pigment phenotype used exclusively animals of one pigment phenotype or the other (wild-type or white axolotls) in their experiments. Accordingly, among those projects there are not only phenotypic variation, which could potencially hide pleotropic effects [37, 38], but also a bias on genetic variation at genome-wide level between these phenotypes, as the association analysis of this study showed.

## Conclusions

Variation is the base of evolution, and its study and characterisation have greatly broadened our knowledge of biological processes. The genetic diversity of species, especially when studying domesticated organisms such as animal models, should be known to ensure the correct interpretation of the results and the reproducibility of any biological experiment. Through the use of high-throughput sequencing technologies, the genetic variation of any organism could be uncovered. For instance, transcriptomic experiments not only provide information about gene expression patterns but also allow the characterisation of the genetic background of the study specimens, as it has been exemplified in this research study.

## Methods

### Sequence data

Data were obtained from the Sequence Read Archive Database, SRA, [39] from all the available projects to the date of data collection. A total of 146 RNA-Seq samples (files downloaded on the 17th of July 2018) from 9 different research projects (6 samples from PRJNA480225, 41 from PRJNA300706, 2 from PRJNA306100, 43 from PRJNA312389, 6 from PRJNA354434, 19 from PRJNA378982, 8 from PRJNA400170, 18 from PRJNA427437 and 3 from PRJNA186654, see Supplementary Table S1 for sample reference ids) were selected based on library strategy (paired-end RNA-Seq from enriched poly-A RNAs) and sequence coverage (more than 10 million reads per sample). The fastq-dump command from SRA Toolkit was used to obtain read sequences from the chosen SRA samples. Each paired read sequence file was aligned to the 23,251 predicted protein-coding genes from the *A. mexicanum* genome assembly v3 [40] using Bowtie 2.3.2 [41] with default parameters. Samtools 1.5 [42] was used to merge and sort the 146 alignment files, after read group information was added to each alignment using Picard tools (http://broadinstitute.github.io/picard/).

### Variant discovery and annotation analysis

Joint variant calling was performed using the HaplotypeCaller tool of GATK 3.6 with a minimum Phred-scaled threshold of 20, following best practices of the GATK program guide [43]. Variant filtration was also carried out according to GATK guidelines, by applying a hard filter to the call set. Variants were filtered using the variant confidence value divided by the unfiltered depth of non-reference samples as a threshold (QD < 2.0) and thinning the data when 3 or more variants were found within 35 bases of each other. Filtered variants were sorted by type of variant, and bi-allelic single nucleotide variants (SNVs) were selected to conduct further analyses after reformatting to flat files (MAP/PED) using VCFtools 0.1.14 [44]. SNVs were characterized by computing several metrics per sample using PLINK 1.9 [45], including: i) genotype missingness, which is the percentage of missing DNA sequence data among loci; ii) allele frequencies; iii) heterozygosity ratios, which is the number of heterozygous sites divided by the number of non-reference/alternative homozygous sites; and iv) transition and transversion rates.

For each analysed protein-coding sequence, the number of SNVs per Kb (frequency of SNVs) was calculated and compared to the overall mean frequency of SNVs across all protein-coding sequences (i.e. number of SNVs per Kb using the total number of bases of all predicted protein-coding genes). Predicted genes were classified using their gene names, and SNV frequencies were scaled subtracting the overall mean frequency for non-redundant genes. The list of non-redundant genes and their scaled SNV frequency was analysed using the statistical enrichment test of the PANTHER webtool with *Homo sapiens* as a reference organism for the annotations sets: GO-Slim Biological Process, Go-Slim Cellular Component, Go-Slim Molecular Function, Pathways, and Protein Class [46]. This test evaluated the likelihood that annotation terms of ranked genes yielded SNV frequency value distributions that deviated significantly from the overall distribution of SNV frequency values.

The base pair positions of SNVs was determined using the predicted protein-coding genes alignments of the axolotl genome assembly v4 [47]. For the wild-caught axolotl samples, unique/private variants were counted and classified by chromosome. Mean wild-caught SNV frequency was calculated relative to the concatenated length of all predicted genes for each chromosome. Chromosomal-level differences in SNV frequency were assessed by comparing SNVs frequencies per chromosome to the overall mean frequency using two-tailed binomial tests at 5% of significance (binom.test) with R 3.3.0 [48].

### Sample diversity and structure analyses

To study relationships between the 146 RNA-Seq samples, multidimensional scaling (MDS) with ten dimensions on a computed matrix of identity by state (IBS) pairwise distances, and estimated kinship coefficients were calculated for SNVs with minor allele frequency (MAF) higher than 0.01 (cut-off for distinguishing common from rare variants) using PLINK 1.9 --cluster, −-matrix, and --mds-plot tool options [45], and VCFtools 0.1.14 --relatedness2 tool option [44], respectively. The subset of SNVs with MAF higher than 0.01 were also used to estimate individual ancestries by cross validation of maximum likelihood searches using Admixture 1.3.0 [49]. Five independent search seeds were used in the Admixture analyses in order to find the most likely number of ancestral populations, K (values tested from 1 to 15). Pairwise F_ST_ (Weir and Cockerham weighted F_ST_) statistics were also calculated between samples of the different projects using VCFtools 0.1.14 --weir-fst-pop tool option [44].

### Association analysis

A subset of SNVs with MAF higher than 0.05 (conservative cut-off for distinguishing common from rare variants) from 38 samples of wild-caught, wild-type and white axolotls (phenotype was designated from their original project, see Supplementary Table S2) was used to perform a basic case/control phenotype-genotype association analysis (two categories: white as a case versus wild/wild-type samples as a control). This analysis was performed using PLINK 1.9 [45] with allow-no-sex option and adjustment for multiple testing using the Benjamini-Hochberg procedure. Variant-phenotype associations were identified using an adjusted *p*-value <1e-5 threshold and their chromosome locations were determined from gene predictions [47]. The variants associated with the study phenotype were classified as synonymous and non-synonymous amino acid changes by exploring the six frames of translation of the axolotl gene predictions (non-synonymous changes were conservatively designated when different amino acids were encoded for all the six open frames). The selection of the 38 samples (19 samples for each category, including one sample of the wild-caught axolotl in the wild/wild-type category) excluded pairs of samples with high kinship coefficients (values lower than 0.0442 for all the pair samples of the wild/wild-type category and for most of the samples of the white category) and population structure. Another phenotype-genotype association analysis of these same 38 samples was performed for a pseudo-random phenotype and no significant variant-phenotype associations were detected (see Supplementary Table S2).

## Supplementary Information

**Additional file 1: Supplementary Material**. The file includes supplementary Tables S1–S9 and supplementary Figures S1–S4. **Table S1**. Sample information. **Table S2**. Sample information for the case/control phenotype-genotype association analysis. **Table S3**. Transitions and transversions in numbers. **Table S4**. Minor allele frequency information for SNVs. **Table S5**. PANTHER enrichment test results. **Table S6**. Dimension information of the multidimensional scaling analysis. **Table S7**. Pairwise FST between projects. **Table S8**. Variants associated to the pigment phenotype. **Table S9**. SNVs per chromosome. **Figure S1**. Multidimensional scaling analysis. **Figure S2**. Likelihood of the admixture analysis for the 146 samples. **Figure S3**. Sample relations. **Figure S4**. Likelihood of the admixture analysis for the 38-sample subset

## Data Availability

The variant calling file built and analysed in this study is available at Open Science Framework (https://osf.io/79m2q/).

## Abbreviations

AGSC: Ambystoma Genetic Stock Centre
Chr: Chromosome
FDR: False discovery rate
FST: Fixation indexes
GO: Gene ontology
INDELs: Insertions and deletions of bases
Kb: Kilobase
MAF: Minor allele frequency
Mb: Megabase
MDS: Multidimensional scaling analysis
SNVs: Single nucleotide variants

## References

[1] Darwin C (1859). On the origins of species by means of natural selection or the preservation of Favoured races in the struggle for life.

[2] Darwin C (1868). The variation of animals and plants under domestication.

[3] Wallace AR (1858). On the tendency of varieties to depart indefinitely from the original type. Zool J Linn Soc-Lond, Zoology.

[4] Andersson L, Georges M (2004). Domestic-animal genomics: deciphering the genetics of complex traits. Nat Re Genet.

[5] Wang GD, Xie HB, Peng MS, Irwin D, Zhang YP (2014). Domestication genomics: evidence from animals. Annu Rev Anim Biosci.

[6] Cieslak M, Reissmann M, Hofreiter M, Ludwig A (2011). Colours of domestication. Biol Rev.

[7] Makino T, Rubin CJ, Carneiro M, Axelsson E, Andersson L, Webster MT (2018). Elevated proportions of deleterious genetic variation in domestic animals and plants. Genome Biol Evol.

[8] Onos KD, Uyar A, Keezer KJ, Jackson HM, Preuss C, Acklin CJ (2019). Enhancing face validity of mouse models of Alzheimer’s disease with natural genetic variation. PLoS Genet.

[9] Crusio WE, Goldowitz D, Holmes A, Wolfer D (2009). Standards for the publication of mouse mutant studies. Genes Brain Behav.

[10] Lithgow GJ, Driscoll M, Phillips P (2017). A long journey to reproducible results. Nature..

[11] Lucanic M, Plummer WT, Chen E, Harke J, Foulger AC, Onken B (2017). Impact of genetic background and experimental reproducibility on identifying chemical compounds with robust longevity effects. Nat Commun.

[12] Milcu A, Puga-Freitas R, Ellison AM, Blouin M, Scheu S, Freschet GT (2018). Genotypic variability enhances the reproducibility of an ecological study. Nat Ecol Evol.

[13] Voelkl B, Vogt L, Sena ES, Würbel H (2018). Reproducibility of preclinical animal research improves with heterogeneity of study samples. PLoS Biol.

[14] Brekke TD, Steele KA, Mulley JF (2017). Inbred or outbred? Genetic diversity in laboratory rodent colonies. G3-Genes Genom Genet.

[15] Reiß C, Olsson L, Hoßfeld U (2015). The history of the oldest self-sustaining laboratory animal: 150 years of axolotl research. J Exp Zool Part B Mol Dev Evol.

[16] Contreras V, Martínez-Meyer E, Valiente E, Zambrano L (2009). Recent decline and potential distribution in the last remnant area of the microendemic Mexican axolotl (*Ambystoma mexicanum*). Biol Conserv.

[17] Voss SR, Woodcock MR, Zambrano L (2015). A tale of two axolotls. BioScience..

[18] Newth DR (1960). Black axolotl, and white. AmSci.

[19] Woodcock MR, Vaughn-Wolfe J, Elias A, Kump DK, Kendall KD, Timoshevskaya N (2017). Identification of mutant genes and introgressed tiger salamander DNA in the laboratory axolotl, *Ambystoma mexicanum*. Sci Rep.

[20] Schaefer KA, Wu W-H, Colgan DF, Tsang SH, Bassuk AG, Mahajan VB (2018). Retraction: Unexpected mutations after CRISPR–Cas9 editing in vivo. Nat Methods.

[21] Brookes AJ (1999). The essence of SNPs. Gene..

[22] Butler MG, Iben JR, Marsden KC, Epstein JA, Granato M, Weinstein BM (2015). SNPfisher: tools for probing genetic variation in laboratory-reared zebrafish. Development..

[23] Doran AG, Wong K, Flint J, Adams DJ, Hunter KW, Keane TM (2016). Deep genome sequencing and variation analysis of 13 inbred mouse strains defines candidate phenotypic alleles, private variation and homozygous truncating mutations. Genome Biol.

[24] Cargill M, Altshuler D, Ireland J, Sklar P, Ardlie K, Patil N (1999). Characterization of single-nucleotide polymorphisms in coding regions of human genes. Nat Genet.

[25] Wang Z, Gerstein M, Snyder M (2009). RNA-Seq: a revolutionary tool for transcriptomics. Nat Rev Genet.

[26] Piskol R, Ramaswami G (2013). Li JB (2013) reliable identification of genomic variants from RNA-seq data. Am J Hum Genet.

[27] Zhang XH, Chasin LA (2006). Comparison of multiple vertebrate genomes reveals the birth and evolution of human exons. Proc Natl Acad Sci.

[28] Liu J, Zhang Y, Lei X (2008). Zhang Z (2008) natural selection of protein structural and functional properties: a single nucleotide polymorphism perspective. Genome Biol.

[29] Ohta T (2000). Mechanisms of molecular evolution. Philos Trans R Soc Lond B Biol Sci.

[30] Kim PM, Korbel JO, Gerstein MB (2007). Positive selection at the protein network periphery: evaluation in terms of structural constraints and cellular context. Proc Natl Acad Sci U S A.

[31] Lowe CB, Kellis M, Siepel A, Raney BJ, Clamp M, Salama SR (2011). Three periods of regulatory innovation during vertebrate evolution. Science..

[32] Komar AA (2007). SNPs, silent but not invisible. Science..

[33] Williams RBH, Chan EKF, Cowley MJ, Little PFR (2007). The influence of genetic variation on gene expression. Genome Res.

[34] Shastry BS. SNPs: impact on gene function and phenotype. In: Komar AA, editor. Single nucleotide polymorphisms methods and protocols. Totowa: Humana Press; 2009. p. 3–22.. 10.1007/978-1-60327-411-1_1 19768584

[35] Liu W, Chen L, Zhang S, Hu F, Wang Z, Lyu J (2019). Decrease of gene expression diversity during domestication of animals and plants. BMC Evol Biol.

[36] Lawson DJ, van Dorp L, Falush D (2018). A tutorial on how not to over-interpret STRUCTURE and ADMIXTURE bar plots. Nat Commun.

[37] Reissmann M, Ludwig A (2013). Pleiotropic effects of coat colour-associated mutations in humans, mice and other mammals. Semin Cell Dev Biol.

[38] Fleck K, Erhardt G, Lühken G (2016). From single nucleotide substitutions up to chromosomal deletions: genetic pause of leucism-associated disorders in animals. Berl Munch Tierarztl Wochenschr.

[39] Leinonen R, Sugawara H, Shumway M (2010). The sequence read archive. Nucleic Acids Res.

[40] Nowoshilow S, Schloissnig S, Fei JF, Dahl A, Pang AWC, Pippel M (2018). The axolotl genome and the evolution of key tissue formation regulators. Nature..

[41] Langmead B, Trapnell C, Pop M, Salzberg S (2009). Ultrafast and memory-efficient alignment of short DNA sequences to the human genome. Genome Biol.

[42] Li H, Handsaker B, Wysoker A, Fennell T, Ruan J, Homer N (2009). (2009) the sequence alignment/map format and SAMtools. Bioinformatics..

[43] Van der Auwera GA, Carneiro MO, Hartl C, Poplin R, del Angel G, Levy-Moonshine A (2013). From fastQ data to high-confidence variant calls: the genome analysis toolkit best practices pipeline. Curr Protoc Bioinformatics.

[44] Danecek P, Auton A, Abecasis G, Albers CA, Banks E, DePristo MA (2011). The variant call format and VCFtools. Bioinformatics..

[45] Purcell S, Neale B, Todd-Brown K, Thomas L, Ferreira MAR, Bender D (2007). PLINK: a tool set for whole-genome association and population-based linkage analyses. Am J Hum Genet.

[46] Mi H, Dong Q, Muruganujan A, Gaudet P, Lewis S, Thomas PD (2009). PANTHER version 7: improved phylogenetic trees, orthologs and collaboration with the gene ontology consortium. Nucleic Acids Res.

[47] Smith JJ, Timoshevskaya N, Timoshevskiy VA, Keinath MC, Hardy D, Voss SR (2019). A chromosome-scale assembly of the axolotl genome. Genome Res.

[48] Development Core Team (2016). R: a language and environment for statistical computing.

[49] Alexander DH, Novembre J (2009). Lange K (2009) fast model-based estimation of ancestry in unrelated individuals. Genome Res.

