# Supplementary material for: Variation under domestication in animal models: the case of the Mexican axolotl
Source: BMC Genomics. 2020 Nov 23;21:827. doi: 10.1186/s12864-020-07248-9 (PMC7685626; doi:10.1186/s12864-020-07248-9)
Supplement: Supplementary file 1 — Additional file 1: Supplementary Material. The file includes supplementary Tables S1–S9 and supplementary Figures S1–S4. Table S1. Sample information. Table S2. Sample information for the case/control phenotype-genotype association analysis. Table S3. Transitions and transversions in numbers. Table S4. Minor allele frequency information for SNVs. Table S5. PANTHER enrichment test results. Table S6. Dimension information of the multidimensional scaling analysis. Table S7. Pairwise FST between projects. Table S8. Variants associated to the pigment phenotype. Table S9. SNVs per chromosome. Figure S1. Multidimensional scaling analysis. Figure S2. Likelihood of the admixture analysis for the 146 samples. Figure S3. Sample relations. Figure S4. Likelihood of the admixture analysis for the 38-sample subset [file 12864_2020_7248_MOESM1_ESM.pdf]

SUPPLEMENTARY MATERIAL

Variation under domestication in animal models: the case of the Mexican axolotl

María Torres-Sánchez

Department of Neuroscience, Spinal Cord and Brain Injury Research Center & Ambystoma Genetic Stock Center, University of Kentucky, Lexington, KY, 40536, United States
Present address: Department of Biology, University of Florida, Gainesville, FL 32611-8525, United States

Author email: torressanchez.maria@gmail.com

This file includes:

Table S1. Sample information
Table S2. Sample information for the case/control phenotype-genotype association analysis
Table S3. Transitions and transversions in numbers
Table S4. Minor allele frequency information for SNVs
Table S5. PANTHER enrichment test results
Table S6. Dimension information of the multidimensional scaling analysis
Table S7. Pairwise F_{ST} between projects
Table S8. Variants associated to the pigment phenotype
Table S9. SNVs per chromosome
Figure S1. Multidimensional scaling analysis
Figure S2. Likelihood of the admixture analysis for the 146 samples
Figure S3. Sample relations
Figure S4. Likelihood of the admixture analysis for the 38-sample subset

Table S1. **Sample information**. For each of the 146 samples, project information, source population (sample pop), phenotype (sample pheno), total number of identified bi-allelic single nucleotide variants (SNVs), number of SNVs with alternative alleles (Non-reference SNVs), percentage of missing genotypes (% missing genotypes), and heterozygosity ratio (Het/Hom) are shown.

Project	Sample Pop	Sample Pheno	Sample ID	Total SNVs	Non-reference SNVs	% missing genotypes	Het/Hom
PRJNA480225	Captive	Wildtype	SRR7499348	313,527	55,952	30.94	1.92
			SRR7499349	324,370	57,608	28.55	1.95
			SRR7499350	230,593	39,546	49.21	1.4
			SRR7499357	262,289	45,235	42.22	1.5
			SRR7499358	319,571	57,627	29.61	1.95
			SRR7499359	287,422	48,610	36.69	1.53
PRJNA300706	Captive	White	SRR2885267	305,785	45,035	32.64	1.14
			SRR2885268	288,250	41,096	36.51	1.05
			SRR2885269	284,121	40,641	37.41	0.87
			SRR2885270	285,281	42,354	37.16	1
			SRR2885271	228,774	32,514	49.61	0.93
			SRR2885273	335,020	51,049	26.20	1.29
			SRR2885274	266,521	39,121	41.30	0.97
			SRR2885275	284,083	41,401	37.42	1.11
			SRR2885283	309,078	45,158	31.92	1.17
			SRR2885284	304,712	45,006	32.88	1.26
			SRR2885285	308,259	42,816	32.10	1.18
			SRR2885286	301,856	39,110	33.51	0.9
			SRR2885287	243,819	33,087	46.29	0.84
			SRR2885288	282,058	41,067	37.87	1
			SRR2885289	249,468	34,673	45.05	0.89
			SRR2885290	217,072	28,288	52.18	0.75
			SRR2885291	273,825	42,176	39.68	1.12
			SRR2885293	327,506	50,455	27.86	1.29
			SRR2885316	316,746	59,258	30.23	1.51
			SRR2885553	369,384	72,137	18.63	2.84
			SRR2885591	354,125	65,794	22.00	2.92
			SRR2885592	281,113	39,498	38.08	1.14
			SRR2885593	291,945	42,715	35.69	1.12
			SRR2885594	290,213	41,107	36.07	1.24
			SRR2885595	299,212	42,384	34.09	1.18

			SRR2885596	297,543	40,727	34.46	1.08
			SRR2885597	328,661	49,254	27.60	1.47
			SRR2885598	311,419	44,374	31.40	1.21
			SRR2885599	315,300	46,699	30.55	1.21
			SRR2885600	309,783	45,412	31.76	1.2
			SRR2885601	292,551	42,989	35.56	1.17
			SRR2885675	320,761	50,604	29.34	1.33
			SRR2885865	359,081	70,011	20.90	3.23
			SRR2885866	355,262	64,996	21.74	3.05
			SRR2885867	116,307	32,075	74.38	1.14
			SRR2885868	270,296	39,247	40.46	0.98
			SRR2885869	290,024	45,860	36.11	1.24
			SRR2885870	285,430	42,260	37.13	1.16
			SRR2885871	301,640	42,050	33.56	1.27
			SRR2885873	309,760	43,471	31.77	1.16
			SRR2885875	289,511	42,019	36.23	1.12
PRJNA306100	Captive	White	SRR3036497	336,404	50,744	25.90	0.63
			SRR3036612	347,007	52,320	23.56	0.65
PRJNA312389	Captive	Unknown	SRR3176172	287,491	54,009	36.67	1.62
			SRR3176173	275,258	49,840	39.37	1.57
			SRR3176174	291,499	55,879	35.79	1.65
			SRR3176176	295,663	53,875	34.87	1.55
			SRR3176177	310,974	57,303	31.50	1.59
			SRR3176178	301,456	54,801	33.60	1.57
			SRR3176179	281,919	50,774	37.90	1.57
			SRR3176180	304,924	56,410	32.83	1.62
			SRR3176181	289,625	52,009	36.20	1.58
			SRR3176184	304,780	55,955	32.86	1.58
			SRR3176185	303,360	56,591	33.18	1.57
			SRR3176186	295,313	53,165	34.95	1.53
			SRR3176187	291,974	53,082	35.68	1.57
			SRR3176188	311,476	57,675	31.39	1.5
			SRR3176189	292,578	52,265	35.55	1.5
			SRR3176190	314,098	58,699	30.81	1.52
			SRR3176191	295,957	54,571	34.81	1.52
			SRR3176192	302,144	56,302	33.44	1.57
			SRR3176193	299,584	54,984	34.01	1.51
			SRR3176196	294,074	54,424	35.22	1.51
			SRR3176197	290,721	53,226	35.96	1.46
			SRR3176198	293,585	53,941	35.33	1.39
			SRR3176199	278,757	49,636	38.60	1.36
			SRR3176200	272,945	47,638	39.88	1.31
			SRR3176201	268,313	47,201	40.90	1.14

			SRR3176202	289,585	52,966	36.21	1.24
			SRR3176203	255,891	44,166	43.63	1.1
			SRR3176204	312,482	56,480	31.17	1.22
			SRR3176205	291,847	51,507	35.71	1.19
			SRR3176208	302,970	55,080	33.26	1.26
			SRR3176209	274,748	47,062	39.48	1.08
			SRR3176210	298,690	53,459	34.21	1.22
			SRR3176215	289,511	51,958	36.23	1.54
			SRR3176216	248,047	43,383	45.36	1.32
			SRR3176217	289,252	51,309	36.28	1.52
			SRR3176218	269,771	49,699	40.58	1.55
			SRR3176219	257,307	43,414	43.32	0.99
			SRR3176220	300,227	52,317	33.87	1.2
			SRR3176221	271,509	47,015	40.19	1
			SRR3176222	328,402	56,120	27.66	1.02
			SRR3176223	382,227	71,212	15.80	1.16
			SRR3176224	367,650	65,497	19.02	1.07
			SRR3176225	327,166	55,140	27.93	0.76
PRJNA354434	Wild	Wild	SRR5042765	228,122	47,372	49.75	0.6
			SRR5042766	249,765	50,193	44.98	0.6
			SRR5042767	189,883	37,335	58.17	0.51
			SRR5042768	278,297	47,532	38.70	0.61
			SRR5042769	252,639	51,172	44.35	0.63
			SRR5042770	228,377	46,182	49.69	0.6
PRJNA378982	Captive	White	SRR5341564	354,483	66,587	21.92	1.6
			SRR5341565	262,724	50,089	42.13	2
			SRR5341568	374,203	70,431	17.57	3.6
			SRR5341569	329,379	43,297	27.45	1.28
			SRR5341570	339,264	45,141	25.27	1.44
			SRR5341571	363,055	49,409	20.03	1.53
			SRR5341572	334,679	44,585	26.28	1.42
			SRR5341573	360,527	61,991	20.58	3.52
			SRR5341574	372,388	53,915	17.97	0.9
			SRR5341575	379,286	57,639	16.45	1.38
			SRR5341576	357,132	70,518	21.33	5.27
			SRR5341577	362,608	71,482	20.13	5.3
			SRR5341578	367,919	73,362	18.96	4.54
			SRR5341579	383,153	75,604	15.60	4.16
			SRR5341580	367,726	65,443	19.00	2.76
			SRR5341581	356,698	64,929	21.43	2.85
			SRR5341582	342,282	56,383	24.60	3.46
			SRR5341583	347,911	61,781	23.36	2.59
			SRR5341584	328,083	48,936	27.73	1.46

PRJNA400170	Captive	White	SRR5974996	298,853	42,745	34.17	1.07
			SRR5974997	302,546	43,618	33.36	1.12
			SRR5974998	303,223	42,260	33.21	1.05
			SRR5974999	303,307	43,254	33.19	1.03
			SRR5975000	307,391	44,099	32.29	1.09
			SRR5975001	301,256	42,507	33.64	1.12
			SRR5975002	303,153	42,895	33.22	1.01
			SRR5975003	297,013	41,477	34.57	1.04
PRJNA427437	Captive	Wildtype	SRR6416913	232,959	36,083	48.68	1.03
			SRR6416914	198,388	28,578	56.30	0.85
			SRR6416915	204,246	30,166	55.01	0.68
			SRR6416916	255,720	39,126	43.67	0.93
			SRR6416917	252,911	37,786	44.29	1.06
			SRR6416918	273,058	43,435	39.85	1.09
			SRR6416919	252,980	40,689	44.27	1.02
			SRR6416920	230,068	34,314	49.32	0.88
			SRR6416921	217,925	31,661	52.00	0.85
			SRR6416922	219,039	32,666	51.75	0.9
			SRR6416923	217,656	31,987	52.06	0.89
			SRR6416924	251,697	38,023	44.56	0.99
			SRR6416925	212,561	31,077	53.18	0.86
			SRR6416926	191,472	26,605	57.82	0.8
			SRR6416927	260,740	42,049	42.56	1
			SRR6416928	269,099	44,418	40.72	1.06
			SRR6416929	195,195	28,621	57.00	0.71
			SRR6416930	63,846	10,408	85.94	0.56
PRJNA186654	Captive	Unknown	SRR650441	299,249	50,641	34.08	1.2
			SRR650442	307,841	52,152	32.19	1.09
			SRR650443	307,841	52,152	32.19	1.09

Table S2. Sample information for the case/control phenotype-genotype association analysis. The table shows the samples and the traits that were used in the genome wide association analyses.

Samples ID	Phenotype	False phenotype
SRR2885267	White	Wild/wildtype
SRR2885273	White	White
SRR2885274	White	Wild/wildtype
SRR2885284	White	White
SRR2885285	White	Wild/wildtype
SRR2885596	White	White
SRR2885865	White	Wild/wildtype
SRR2885866	White	White
SRR2885867	White	Wild/wildtype
SRR2885873	White	White
SRR3036497	White	Wild/wildtype
SRR5341565	White	White
SRR5341568	White	Wild/wildtype
SRR5341573	White	White
SRR5341581	White	Wild/wildtype
SRR5341582	White	White
SRR5341583	White	Wild/wildtype
SRR5341584	White	White
SRR5975000	White	Wild/wildtype
SRR5042768	Wild/wildtype	White
SRR6416913	Wild/wildtype	Wild/wildtype
SRR6416914	Wild/wildtype	White
SRR6416915	Wild/wildtype	Wild/wildtype
SRR6416916	Wild/wildtype	White
SRR6416917	Wild/wildtype	Wild/wildtype
SRR6416918	Wild/wildtype	White
SRR6416919	Wild/wildtype	Wild/wildtype
SRR6416920	Wild/wildtype	White
SRR6416921	Wild/wildtype	Wild/wildtype
SRR6416922	Wild/wildtype	White
SRR6416923	Wild/wildtype	Wild/wildtype
SRR6416924	Wild/wildtype	White
SRR6416925	Wild/wildtype	Wild/wildtype
SRR6416926	Wild/wildtype	White
SRR6416927	Wild/wildtype	Wild/wildtype

SRR6416928	Wild/wildtype	White
SRR6416929	Wild/wildtype	Wild/wildtype
SRR6416930	Wild/wildtype	White

Table S3. Transitions and transversions in numbers. SNVs classification based on the change from the reference allele to the alternative allele.

Transitions				Transversions							
C/T	T/C	G/A	A/G	C/A	A/C	G/T	T/G	C/G	G/C	T/A	A/T
82,766	82,478	54,970	62,389	22,519	18,810	27,722	20,449	13,337	20,822	20,311	27,400

Table S4. Minor allele frequency (MAF) information for SNVs.

**MAF**	**< 0.01**	**0.01–0.05**	**0.05–0.1**	**0.1–0.2**	**0.2–0.3**	**0.3–0.4**	**0.4–0.5**	**0.5**
SNVs	202,039	96,254	27,095	40,848	30,858	28,546	24,862	3,471
%	45.36	20.01	6.12	9.06	6.83	6.38	5.51	0.735

Table S5. PANTHER enrichment test results.

Annotation set	Term	Term description	Enrichment direction	Number of annotated gene predictions	FDR
Biological process	GO:0035249	Synaptic transmission, glutamatergic	-	24	3.66E-03
Biological process	GO:0043170	Macromolecule metabolic process	-	1314	1.38E-02
Biological process	GO:0032501	Multicellular organismal process	-	599	2.35E-02
Biological process	GO:0007215	Glutamate receptor	-	25	2.23E-02

		signaling pathway			
Biological process	GO:0010467	Gene expression	-	989	2.78E-02
Cellular component	GO:0044445	Cytosolic part	-	62	3.10E-05
Cellular component	GO:0022626	Cytosolic ribosome	-	39	4.19E-05
Cellular component	GO:0034703	Cation channel complex	-	43	3.37E-04
Cellular component	GO:0045211	Postsynaptic membrane	-	30	3.85E-04
Cellular component	GO:0005887	Integral component of plasma membrane	-	335	5.62E-04
Cellular component	GO:0022625	Cytosolic large ribosomal subunit	-	24	5.26E-04
Cellular component	GO:0034702	Ion channel complex	-	53	9.48E-04
Cellular component	GO:0098794	Postsynapse	-	41	9.93E-04
Cellular component	GO:0097458	Neuron part	-	226	1.35E-03
Cellular component	GO:0000502	Proteasome complex	-	25	1.24E-03
Cellular component	GO:1902495	Transmembrane transporter complex	-	54	1.38E-03
Cellular component	GO:0014069	Postsynaptic density	-	29	6.46E-03
Cellular component	GO:0005829	Cytosol	-	352	9.67E-03
Cellular component	GO:0036477	Somatodendritic compartment	-	77	1.08E-02
Cellular component	GO:0005839	Proteasome core complex	-	10	1.15E-02
Cellular component	GO:0031224	Intrinsic component of membrane	-	448	1.43E-02

Cellular component	GO:0016021	Integral component of membrane	-	444	1.44E-02
Cellular component	GO:0031256	Leading edge membrane	-	11	2.00E-02
Cellular component	GO:0005634	Nucleus	-	1144	2.07E-02
Cellular component	GO:0044430	Cytoskeletal part	-	30	2.36E-02
Cellular component	GO:0030425	Dendrite	-	60	2.77E-02
Cellular component	GO:0005746	Mitochondrial respiratory chain	-	12	4.41E-02
Molecular function	GO:0051020	GTPase binding	+	112	1.94E-04
Molecular function	GO:0060589	Nucleoside-triphosphatase regulator activity	+	59	2.42E-03
Molecular function	GO:0005096	Gtpase activator activity	+	52	2.47E-03
Molecular function	GO:0017016	Ras gtpase binding	+	66	4.87E-03
Molecular function	GO:0031267	Small gtpase binding	+	67	4.78E-03
Molecular function	GO:0030695	Gtpase regulator activity	+	54	4.79E-03
Molecular function	GO:0005085	Guanyl-nucleotide exchange factor activity	+	53	7.23E-03
Molecular function	GO:0052745	Inositol phosphate phosphatase activity	+	5	3.22E-02
Molecular function	GO:0017137	Rab gtpase binding	+	38	4.51E-02
Molecular function	GO:1901363	Heterocyclic compound binding	-	864	1. 95E-06
Molecular function	GO:0003676	Nucleic acid binding	-	838	5.88E-06
Molecular function	GO:0003723	RNA binding	-	276	2.04E-05

Molecular function	GO:0005249	Voltage-gated potassium channel activity	-	34	9.77E-05
Molecular function	GO:0003735	Structural constituent of ribosome	-	53	1.93E-04
Molecular function	GO:0005267	Potassium channel activity	-	53	2.15E-04
Molecular function	GO:0036094	Small molecule binding	-	127	1.96E-04
Molecular function	GO:0042165	Neurotransmitter binding	-	40	1.84E-04
Molecular function	GO:0022803	Passive transmembrane transporter activity	-	90	2.18E-04
Molecular function	GO:0015267	Channel activity	-	90	1.98E-04
Molecular function	GO:0022836	Gated channel activity	-	87	3.46E-04
Molecular function	GO:0022834	Ligand-gated channel activity	-	67	3.47E-04
Molecular function	GO:0015276	Ligand-gated ion channel activity	-	67	3.52E-04
Molecular function	GO:0008066	Glutamate receptor activity	-	26	8.89E-04
Molecular function	GO:0016595	Glutamate binding	-	26	8.34E-04
Molecular function	GO:0030594	Neurotransmitter receptor activity	-	46	9.18E-04
Molecular function	GO:0043177	Organic acid binding	-	47	9.17E-04
Molecular function	GO:0031406	Carboxylic acid binding	-	47	8.69E-04
Molecular function	GO:0022890	Inorganic cation transmembrane transporter activity	-	207	1.60E-03
Molecular function	GO:0016597	Amino acid binding	-	36	2.23E-03
Molecular function	GO:0005488	Binding	-	2461	3.18E-03
Molecular	GO:0022857	Transmembrane	-	403	3.40E-03

function		transporter activity			
Molecular function	GO:0003700	DNA-binding transcription factor activity	-	433	4.62E-03
Molecular function	GO:0008324	Cation transmembrane transporter activity	-	220	4.78E-03
Molecular function	GO:0004930	G-protein coupled receptor activity	-	168	5.21E-03
Molecular function	GO:0003677	DNA binding	-	507	5.22E-03
Molecular function	GO:0004016	Adenylate cyclase activity	-	63	5.68E-03
Molecular function	GO:0000976	Transcription regulatory region sequence-specific DNA binding	-	189	5.92E-03
Molecular function	GO:0015079	Potassium ion transmembrane transporter activity	-	68	7.84E-03
Molecular function	GO:0003729	Mrna binding	-	76	8.62E-03
Molecular function	GO:0044212	Transcription regulatory region DNA binding	-	216	1.04E-02
Molecular function	GO:0016849	Phosphorus-oxygen lyase activity	-	67	1.08E-02
Molecular function	GO:0004888	Transmembrane signaling receptor activity	-	241	1.08E-02
Molecular function	GO:0005230	Extracellular ligand-gated ion channel activity	-	46	1.27E-02
Molecular function	GO:0000981	RNA polymerase II transcription factor activity,	-	168	1.61E-02

		sequence-specific DNA binding			
Molecular function	GO:0015075	Ion transmembrane transporter activity	-	274	2.02E-02
Molecular function	GO:0140110	Transcription regulator activity	-	506	2.68E-02
Molecular function	GO:0005251	Delayed rectifier potassium channel activity	-	10	4.32E-02
Pathways	P00047	PDGF signaling pathway	+	100	1.03E-02
Protein Class	PC00022	G-protein modulator	+	172	2.70E-04
Protein Class	PC00113	Guanyl-nucleotide exchange factor	+	52	4.66E-02
Protein Class	PC00031	RNA binding protein	-	310	2.33E-09
Protein Class	PC00171	Nucleic acid binding	-	817	1.20E-07
Protein Class	PC00202	Ribosomal protein	-	71	1.61E-07
Protein Class	PC00119	Homeodomain transcription factor	-	47	3.23E-04
Protein Class	PC00021	G-protein coupled receptor	-	109	5.06E-03
Protein Class	PC00116	Helix-turn-helix transcription factor	-	93	2.44E-02
Protein Class	PC00218	Transcription factor	-	554	3.21E-02
Protein Class	PC00224	Translation initiation factor	-	35	4.25E-02

Table S6. Dimension information of the multidimensional scaling analysis. Table shows the explained variance percentage of the genetic distances for each dimension.

**MDS**	**1**	**2**	**3**	**4**	**5**	**6**	**7**	**8**	**9**	**10**
Variance %	37.93	18.75	8.56	7.69	6.05	5.75	4.97	3.72	3.70	2.88

Table S7. Pairwise Weir and Cockerham weighted F_{ST} estimate between projects.

	PRJNA 480225	PRJNA3 00706	PRJNA3 06100	PRJNA3 12389	PRJNA3 54434	PRJNA3 78982	PRJNA4 00170	PRJNA4 27437	PRJNA1 86654
PRJNA480225									
PRJNA300706	0.152								
PRJNA306100	0.368	0.248							
PRJNA312389	0.325	0.274	0.416						
PRJNA354434	0.470	0.373	0.664	0.495					
PRJNA378982	0.178	0.087	0.275	0.279	0.388				
PRJNA400170	0.262	0.128	0.420	0.362	0.532	0.155			
PRJNA427437	0.168	0.156	0.250	0.302	0.369	0.164	0.215		
PRJNA186654	0.376	0.266	0.423	0.413	0.637	0.280	0.426	0.279	

Table S8. Variants associated to the pigment phenotype. The table shows the results of the variant-phenotype association analysis with the referent (Ref) and alternative (Alt) alleles, position of the SNVs, and their change on the amino-acid sequence (category of change: NS = non-synonymous for the six-frames, S = synonymous).

**Gene prediction**	**SNV position**	**Ref/ Alt**	**Category of change**	**Chromosome/Scaffold location of the gene prediction**	**Adjusted p-value**
ppp1r32	1767	T/A	4S	chr11:608104027-608567284	4.724e-06
prkag2	1988	G/A	1/4/6S	chr2Q:669815997-671905509	5.966e-08
edn3	1163	C/G	NS	chr3P:323871542-324380667	7.917e-06

edn3	784	C/T	1/4S	chr3P:323871542-324380667	3.159e-07
pxk	1564	C/G	NS	chr2P:395883508-396811984	2.747e-06
pxk	211	T/G	1/4S	chr2P:395883508-396811984	7.917e-06
schip1	978	C/T	1/4S	chr10:893144336-893762530	7.917e-06
tbc1d5	1718	C/T	1/4S	chr2Q:1460216965-1463041082	3.683e-06
loc108709264	655	A/G	1/4S	chr7:190875115-191205544	5.663e-06
nuf2.l	167	C/T	1/4/5S	chr1Q:625846185-626394876	2.747e-06
tex26.s	661	G/A	1/4S	chr7:1627232870-1627431340	2.747e-06
fbln1	5051	G/C	4S	chr12:143306565-144207373	4.772e-06
emb	794	G/A	4S	chr6P:424997966-425053153	2.41e-06
bcas1	2351	C/T	1/4S	chr3P:428956033-429713589	4.724e-06
bcas1	2485	G/C	NS	chr3P:428956033-429713589	7.917e-06
loc108717189	2808	T/C	1/4S	AMEXG_0030042853:31752-35880	2.747e-06
gmeb2	2247	T/G	1S	chr3P:290222210-290417327	1.411e-06
ldb3	1848	C/A	NS	chr8:901925629-902602771	7.613e-06
ldb3	8477	A/T	1/4S	chr8:901925629-902602771	8.888e-06
mydgf	199	T/C	1/4S	chr1P:430185034-430263334	2.747e-06
draxin	1015	C/A	1S	chr8:372047655-372268841	7.917e-06
sfrp2	744	A/T	1S	AMEXG_0030069366:52188-64073	2.823e-06
fbn3	2577	A/T	1/4S	chr1P:561988136-562434466	7.778e-06
fbn3	373	A/G	1/4S	chr1P:561988136-562434466	7.917e-06
fads2	2426	A/G	1/4S	chr11:585940342-586180132	7.917e-06
enpp4	1154	G/A	1/4S	chr1Q:1326446928-1326862220	8.225e-06
rapgefl1	3971	A/G	1/2/4S	chr13:587306608-587670423	1.658e-06
sec62	4378	G/A	1/4S	chr10:825284344-825902012	5.099e-06
sec62	6988	T/C	1/4S	chr10:825284344-825902012	1.337e-06
sec62	8505	G/A	1/4S	chr10:825284344-825902012	3.141e-06
loc106732794	1415	G/C	NS	chr2Q:815916106-815946964	5.574e-06
AMEXTC_0340000052 082_hypothetical	1940	G/A	1/4S	chr3P:1048717572-1049210713	7.76e-06
AMEXTC_0340000052 082_hypothetical	2181	A/G	4S	chr3P:1048717572-1049210713	2.747e-06
loc108803380	2868	C/G	1S	chr4P:39184273-39285262	4.717e-07
epb41l5	670	T/C	1/4S	chr9:1304775680-1304820493	2.747e-06
colec12	2490	C/T	1/4S	chr5Q:409099168-409650868	2.747e-06
mrps30	939	A/G	1/4S	chr6P:398620686-398781682	9.199e-06
gna12	1475	T/A	1/4S	chr2Q:521821448-522245718	1.002e-06
gna12	1807	T/C	1/4S	chr2Q:521821448-522245718	6.961e-07
gna12	1933	T/C	1S	chr2Q:521821448-522245718	6.961e-07
hagh.l	1649	A/C	1/4S	chr2Q:369691264-370696987	4.242e-06
hagh.l	1698	T/G	1/6S	chr2Q:369691264-370696987	4.242e-06
hagh.l	2031	T/G	4S	chr2Q:369691264-370696987	5.099e-06
hagh.l	2198	T/C	1/4S	chr2Q:369691264-370696987	2.747e-06

hagh.l	2355	C/G	1S	chr2Q:369691264-370696987	2.747e-06
hagh.l	2749	C/T	1S	chr2Q:369691264-370696987	4.242e-06
hagh.l	2808	T/G	NS	chr2Q:369691264-370696987	2.747e-06
nsfl1c	3133	T/G	4S	chr3P:286539226-286876543	5.966e-08
naa38	1407	G/A	4S	chr1P:146506350-146577474	7.917e-06
loc104535489	346	A/G	4S	chr1P:20073952-20105783	7.917e-06
brwd3	3094	T/C	1/4S	chr5P:467547013-468511289	9.581e-06
dnm2	3098	G/A	1/4S	chr3Q:1208857193-1210413618	5.099e-06
kcnmb1.l	473	A/G	1/4S	chr3Q:520908973-521023185	7.917e-06
syt11	877	A/C	1/3/4S	chr1P:644909903-645154969	4.724e-06
loc100619372	2915	A/T	1/4S	chr1P:41907577-42043554	1.658e-06
grk5	1525	G/A	1/4S	chr8:1649089400-1649679326	7.46e-06
lipe	2794	A/G	1/4S	chr1Q:501465118-501801810	3.159e-07
lipe	4862	T/C	1/4S	chr1Q:501465118-501801810	7.46e-06
etfbkmt	477	G/C	1/4S	chr12:347100581-347377062	1.411e-06
tmprss2	1093	G/T	4S	chr7:145549736-146123990	7.917e-06
tnfaip8	1023	T/C	1/4S	chr6P:914997506-915739389	3.159e-07
loc108702636	1503	T/C	1/4S	chr3P:322593828-323564837	3.159e-07
loc108702636	1586	G/A	1/4S	chr3P:322593828-323564837	4.914e-06
loc108702636	214	C/G	4S	chr3P:322593828-323564837	3.457e-06
loc108702636	2185	T/G	NS	chr3P:322593828-323564837	7.917e-06
loc108702636	2263	A/T	NS	chr3P:322593828-323564837	2.747e-06
loc108702636	2372	A/C	1/4S	chr3P:322593828-323564837	3.841e-07
loc108702636	2919	G/C	NS	chr3P:322593828-323564837	4.724e-06
loc103760181	1433	A/C	NS	chr10:196795242-196865392	4.242e-06
oxct1	1817	C/T	1/4S	chr6P:362601706-363578016	2.028e-06
glucocorticoid	2905	T/G	NS	chr3P:1043770765-1044417969	3.62e-06
glucocorticoid	4035	A/T	1S	chr3P:1043770765-1044417969	4.772e-06
glucocorticoid	4076	T/C	1/4S	chr3P:1043770765-1044417969	6.337e-07
loc102357877	7416	A/G	4S	chr3Q:858701399-859140518	2.747e-06
mapre1.l	1090	T/C	1/4S	chr3P:317528476-317742389	3e-06
rnf11.l	663	C/T	1S	chr1Q:1262683084-1262898442	6.337e-07
rpl21	590	C/T	1/4S	chr7:1629688586-1629779720	3.159e-07
rad50	1187	G/T	1/4S	chr3P:1072428032-1073736315	4.517e-07
rad50	2896	T/C	1/4S	chr3P:1072428032-1073736315	3.138e-06
rad50	2953	A/C	1S	chr3P:1072428032-1073736315	3.605e-06
rad50	3232	C/T	1/4S	chr3P:1072428032-1073736315	4.604e-06
cunh5orf51	822	T/A	1S	chr6P:361765979-362273600	5.49e-06
cunh5orf51	957	T/A	1S	chr6P:361765979-362273600	1.411e-06
loc102366839	7129	C/T	1/3/4S	chr2P:1098104594-1099674174	7.917e-06
tmed1	1564	G/T	NS	chr3Q:1220879867-1220887029	5.099e-06
tmed1	2342	C/T	1/4S	chr3Q:1220879867-1220887029	7.917e-06
tmed1	4621	G/T	NS	chr3Q:1220879867-1220887029	4.724e-06

loc110078335	206	C/T	1/4S	chr12:476811940-476815002	1.417e-06
top1.2	345	C/G	4S	chr1Q:219338924-219710363	7.917e-06
fundc2	2088	T/A	NS	chr2Q:1478400036-1478537149	4.772e-06
srgap2	2816	A/C	1/3/4S	chr13:37492710-38046280	3.138e-06
srgap2	3529	C/T	1/4S	chr13:37492710-38046280	4.772e-06
farsa	1411	A/G	1/4S	chr3Q:1187539863-1187699121	7.613e-06
farsa	1766	A/T	1/4S	chr3Q:1187539863-1187699121	7.613e-06
farsa	1854	T/G	NS	chr3Q:1187539863-1187699121	4.242e-06
farsa	2225	C/T	1S	chr3Q:1187539863-1187699121	3.605e-06
farsa	2375	T/C	NS	chr3Q:1187539863-1187699121	7.613e-06
farsa	2443	T/C	1/4S	chr3Q:1187539863-1187699121	7.917e-06
farsa	2475	A/T	4S	chr3Q:1187539863-1187699121	7.917e-06
farsa	266	C/G	1/4S	chr3Q:1187539863-1187699121	6.224e-06
farsa	561	T/G	4S	chr3Q:1187539863-1187699121	7.917e-06
znf281	280	C/A	1/4S	chr13:372674911-372811418	4.724e-06
mRNA00395	2711	A/G	1/4S	chr4Q:1109221278-1109226951	4.416e-06
mRNA01262	643	T/A	4S	chr5Q:150156043-150157440	7.917e-06

Table S9. SNVs per chromosome.

**Chromosome**	**Total SNVs**	**Private wild-specific SNVs**	**Binomial test p value**
1	56,431	3,145	0.2411
2	47,392	2,860	0.001269
3	49,353	3,097	3.03E-08
4	29,922	1,545	7.22E-05
5	28,712	1,574	0.1328
6	38,860	2,292	0.07605
7	26,452	1,330	2.443e-06
8	26,933	1,597	0.08971
9	29,052	1,566	0.02844
10	25,124	1,400	0.4376
11	24,987	1,476	0.1366
12	20,823	1,076	0.001051
13	20,928	1,326	6.75E-05
14	13,227	763	0.6934
Unknown	15,777	778	-

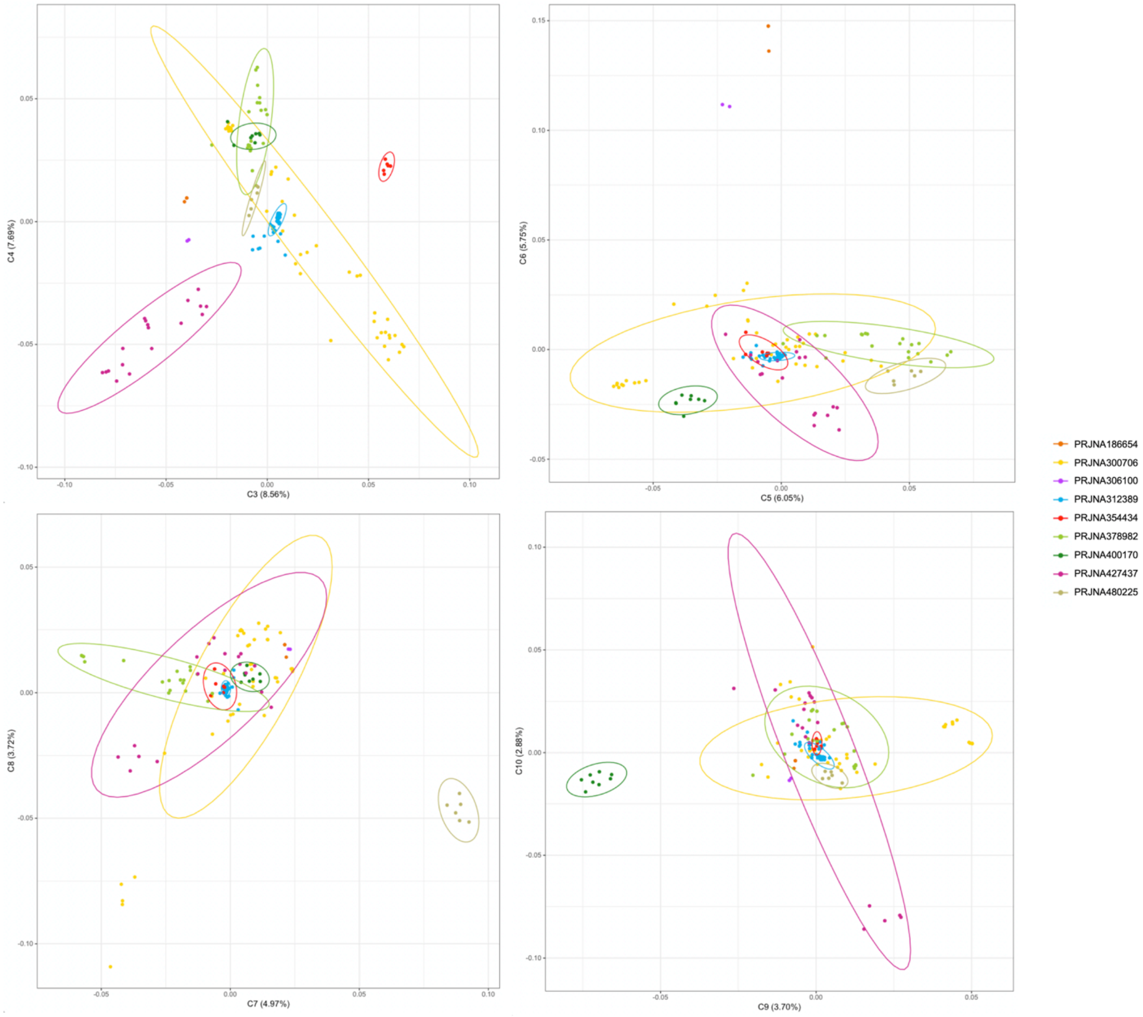

Figure S1. Multidimensional scaling analysis. Scatterplots represent the dimensions 3 to 10 of the multidimensional scaling analysis. The samples of each project are symbolised by different colors and grouped by elipses of the same color.

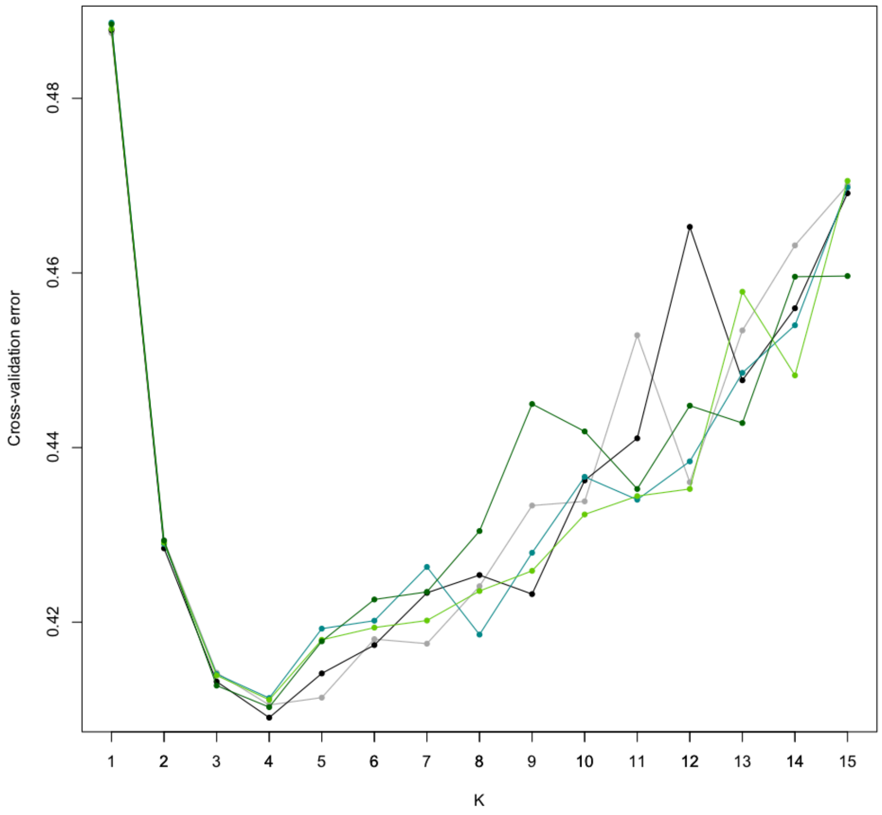

Figure S2. **Likelihood of the admixture analysis for the 146 samples**. Five independent search seeds of the admixture analysis are represented along with their cross-validation error value for the fifteen tested clusters (K) of ancestral populations.

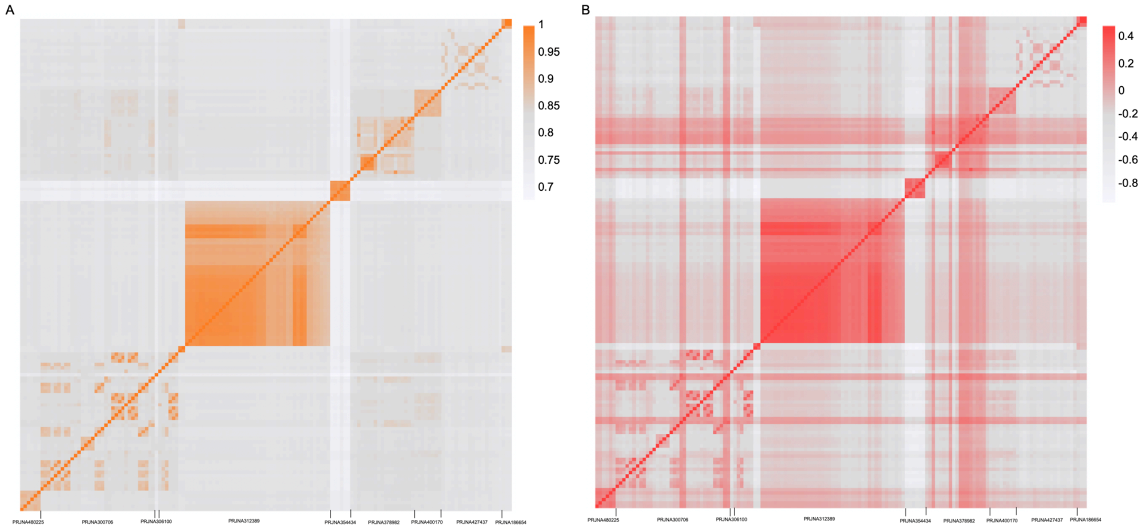

Figure S3. Relationships among samples. (A) Heatmap of the identity by state (IBS) pairwise distances and (B) heatmap of the identity by descendant (IBD) pairwise distances between samples. The data is displayed in a grid where each row and column represent a sample. In the diagonal, the distance between one sample and itself is represented. Labels on the x axis display project ids.

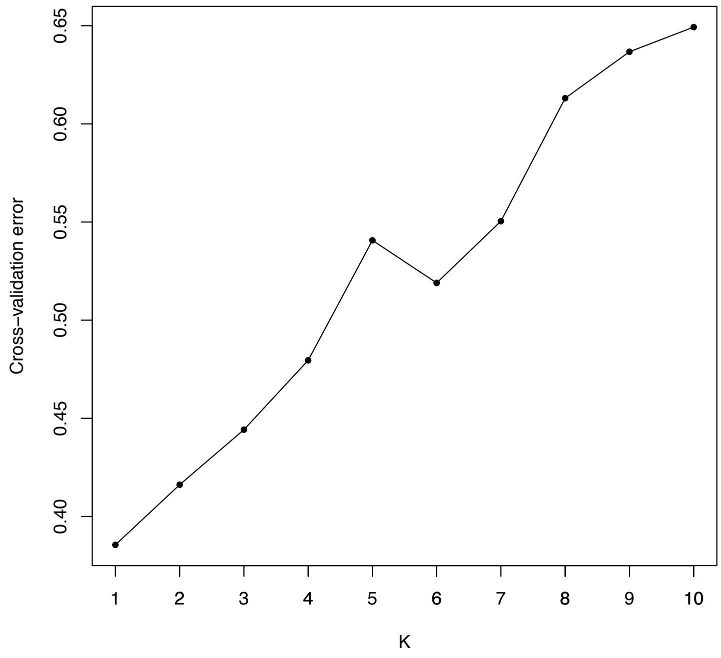

Figure S4. Likelihood of the admixture analysis for the 38-sample subset. Cross-validation error values for the ten tested clusters (K) of ancestral populations are represented in the plot.
